# ERCP-induced perforation: review and revisit after half a century

**DOI:** 10.12688/f1000research.129637.2

**Published:** 2024-03-15

**Authors:** Abdel Rahman A. A. Al Manasra, Zaid Mesmar, Tarek Manasreh, Hanan M . Hammouri, Anas Husein, Khaled Jadallah, Mohammed Bani hani, Niazy Abu Farsakh, Shatha K. Shahwan, Doaa Al-qaoud, Jehad Fataftah

**Affiliations:** 1 Department of general surgery and urology, faculty of medicine, Jordan University of Science and Technology, Iribid, Irbid, 22110, Jordan; 2 Department of mathematics and statistics, Jordan University of Science and Technology, Irbid, Irbid, 22110, Jordan; 3 Department of internal medicine, faculty of medicine, Jordan University of Science and Technology, Irbid, Irbid, 22110, Jordan; 4 Department of pediatrics, Faculty of medicine, The Hashemite university, Zarqa, Zarqa, 13133, Jordan; 5 Department of radiology, Faculty of medicine, The Hashemite university, Zarqa, Zarqa, 13133, Jordan

**Keywords:** ERCP, duodenal perforation, Stapfer classification, Obstructive jaundice, Choledocholithiasis

## Abstract

**Background:**

Endoscopic retrograde cholangiopancreatography (ERCP) is an invasive procedure. We aimed to investigate ERCP-induced perforations at our institution and conduct a comprehensive review of literature on ERCP-induced perforations (EIP) since the introduction of this procedure as a therapeutic intervention.

**Methods:**

This was a case-control study, in which charts of all patients diagnosed with ERCP-induced duodenal perforation were reviewed and compared to a control group without perforation. Patient’s sociodemographic and clinical data, including ERCP procedure-related data, were gathered.

**Results:**

A total of 996 ERCP procedures were performed; only 13 patients proved to have EIP. Obstructive jaundice was the most common indication for ERCP. The main predisposing factor was difficult cannulation (P = 0.003). In total, five patients required surgical treatment; the majority of them had type I perforation, whereas type IV was the most common in patients who were treated conservatively. The overall mortality rate was 15%, the surgical group had a slightly higher mortality rate.

**Conclusions:**

Fifty years after the introduction of ERCP for therapy, it remains an invasive procedure that carries significant morbidity and mortality, even in skilled hands or at high- volume units. Conservative management of perforation yields favorable outcomes in selected patients.

## Introduction

Endoscopic retrograde cholangiopancreatography (ERCP) is one of the most commonly utilized procedures for both diagnostic and therapeutic indications. It was first developed in clinical practice in 1968 as a diagnostic tool. ^1^ Cannulation of the ampulla and sphincterotomy were introduced in the early 1970s with consequent evolution of ERCP into a therapeutic procedure. ^1^^,^^2^ Nowadays ERCP is almost exclusively indicated for the therapy of hepatobiliary disorders since other less invasive diagnostic modalities (for example: magnetic resonance cholangiopancreatography (MRCP)) which possess similar sensitivity, are widely available.

In spite of the valuable contribution of ERCP in patients’ management, it is not a complication-free intervention. The ERCP-related morbidity was estimated to be 6.9%; it also carries a mortality rate of 0.33%. ^3^ The spectrum of complications includes pancreatitis being the most common, followed by cholangitis, bleeding and perforation. The incidence of severe complications is approximately four times higher in ERCP than in other endoscopic procedures. ^4^

One of the most feared complications is ERCP-induced perforation (EIP). Although EIP is uncommon, it is potentially fatal. Moreover, the treatment of such complication is still controversial.

This study represents the first analysis of EIPs in Jordan. Along with this analysis, we aimed to conduct a comprehensive review of literature for ERCP-induced perforations since the introduction of this procedure as a therapeutic intervention.

## Methods

### Study design

This was a retrospective study in which gastroenterology, surgery, anesthesiology, and intensive care data were collected from the electronic medical charts of patients who underwent ERCP during the period of January 2016 to June 2020.

The following information was extracted: patient demographics, comorbidities, indication for endoscopy, ERCP findings, presumed risk factors for difficult procedure (sphincterotomy *versus* precut, dilatation procedures, failed cannulation and relevant past anatomy-altering operation). In addition, clinical presentation of perforation, radiologic findings, time to diagnosis, as well as perforation type according to the Stapfer classification ^5^ (Type I: lateral – free wall – or medial duodenal wall perforation, type II: peri-Vaterian injuries, type III: distal bile duct or pancreatic duct perforation, type IV: retroperitoneal air alone) were all documented. The treatment approach (conservative or operative), need for additional interventions ( *e.g.* percutaneous drainage), length of hospital stay (LOS), morbidity, and mortality are also reported.

Sex and gender differences between participants were not taken into consideration, because in this study, the choice of design is little affected by implications of sex and gender. The sex of participants was defined based on self-report.

### Inclusion criteria

Patients who were diagnosed to have endoscopic perforation (EP) either during ERCP or by imaging studies such as computed tomography (CT) were included. As a control group, 43 patients without ERCP-related complications were randomly selected from the same time period. The randomization process was done by selecting a control patient from every 23 patients after arranging the 966 patients chronologically by ERCP date.

### Ethics

The study protocol was reviewed and approved by the Institutional Review Board at King Abdullah University Hospital and by the Committee of Research on Human Subjects at the Jordan University of Science and Technology (Non-funded research No: 20200428). Patients’ data were kept anonymous and confidential. All study procedures were conducted according to the World Medical Association Declaration of Helsinki.

### Procedures

The management plan was dictated by the attending surgeon on call. Patients who were treated conservatively were kept nil per OS and received intravenous fluids, broad spectrum antibiotics and placement of a nasogastric tube. Failure to improve with conservative management, alarming CT findings and the presence of diffuse peritonitis were considered indications for operative treatment. Patients received percutaneous drainage for intra-abdominal collections and enteral or parenteral feeding as appropriate.

### Statistical analysis

Data were managed and analyzed using JMP (version 15). Statistical summaries were presented. Q-Q plots were used to examine normality assumption for continuous variables. Afterward, significant differences in participants’ characteristics were analyzed using a t-test, Fisher exact test or logistic regression. Additionally, odds ratios were used for significant results. Alpha was set at 0.05.

## Results

A total of 996 ERCP procedures were performed by three experienced endoscopists (board certified gastroenterologist, licensed to perform endoscopy after training in an accredited structured ERCP program). Thirteen patients (1.34%) were diagnosed with EIP. Additionally, 43 other patients who also underwent ERCP, but with no endoscopic induced perforation were randomly selected as a control group, all patients had the procedure for the first time ( Table 1).

**Table 1.** Patients characteristics.

Characteristics	Non-EIP (n = 43)	EIP (n = 13)	P value
Sex - female (%)	20 (47%)	9 (69%)	^d^NS
Age (mean ± ^e^SD)	52.60 ± 20.48	48.77 ± 15.27	NS
Comorbidities			
^a^HTN - n (%)	17 (47%)	9 (69%)	NS
^b^DM - n (%)	15 (22.5%)	7 (18.9%)	NS
^c^IHD - n (%)	2 (2%)	0 (0%)	NS
Past relevant surgery - n (%)	9 (21%)	2 (15%)	NS
Laparoscopic cholecystectomy - n (%)	7 (16%)	2 (15%)	NS
Hydatid cyst resection - n (%)	1 (2%)	0 (0%)	NS
Whipple procedure - n (%)	1 (2%)	0 (0%)	NS
Relevant Neoplasm	8 (19%)	2 (15%)	NS
Pancreatic carcinoma - n (%)	3 (7%)	1 (8%)	NS
Cholangiocarcinoma- n (%)	4 (9%)	1 (8%)	NS
Gastric carcinoma - n (%)	1 (2%)	0 (0%)	NS

^a^ HTN, hypertension.

^b^ DM, diabetes mellitus.

^c^ IHD, ischemic heart disease.

^d^ NS, not Significant.

^e^ SD, standard deviation.

EIP group: There were nine women (69%) and four men (31%) with a mean age of 52.6 years. Approximately two-thirds (69%) of patients had hypertension, 18.9% had diabetes mellitus while none were diagnosed to have ischemic heart disease. Only two patients (15%) had a history of abdominal surgery, both of which were laparoscopic cholecystectomy. One patient had pancreatic adenocarcinoma and another patient was known to have cholangiocarcinoma.

*ERCP indications*

By far, the most common indication for ERCP was obstructive jaundice (61%), followed by choledocholithiasis (23%). Other indications included gallstone pancreatitis and bile leak after cholecystectomy ( Table 2).

**Table 2.** ERCP indications and characteristics.

	Non- EIP (n = 43)	^d^EIP (n = 13)	P value
Indications			
Choledocholithiasis - n (%)	12 (28%)	3 (23%)	NS
Cholangitis - n (%)	2 (4%)	0 (0%)	NS
Obstructive jaundice - n (%)	23 (53%)	8 (61%)	NS
Gallstone pancreatitis - n (%)	5 (11%)	1 (7%)	NS
Leak after cholecystectomy - n (%)	1 (2%)	1 (7%)	^c^NA
Characteristics			
Dilated ^a^CBD - n (%)	20 (46%)	4 (31%)	^b^NS
Filling defect - n (%)	11 (26%)	2 (15%)	NS
Papillotomy - n (%)	34 (79%)	11 (85%)	NS
CBD stricture balloon dilatation - n (%)	29 (67%)	4 (31%)	.0214
Stone extraction - n (%)	15 (19%)	3 (23%)	NS
Sludge extraction - n (%)	13 (30%)	4 (31%)	NS
Stent placement - n (%)	14 (33%)	2 (15%)	NS
Pus - n (%)	1 (2%)	0 (0%)	NS
Failure of cannulation - n (%)	3 (6%)	6 (46%)	.003
Premature termination of procedure - n (%)	3 (6%)	6 (46%)	.003

^a^ CBD, Common bile duct.

^b^ NS, not significant.

^c^ not applicable.

^d^ EIP, Endoscopic Induced Perforation.

*ERCP characteristics*

Dilated common bile duct (CBD) was observed in 31% of cases and 15% had a filling defect. Precut papillotomy was performed for 85% of the EIP group, 23% had a stone extracted during ERCP. Failure of cannulation was experienced in half of patients (six patients, 46%).

### Risk factors for perforation

*Patient’s characteristics*

Notably, a higher number of patients had hypertension in the EIP group compared to the non-EIP group (69% *versus* 47%) but fewer had diabetes mellitus (18.9% *versus* 22.5%) with no statistical significance ( Table 1).

*Indications for ERCP*

The presence of obstructive jaundice and post-cholecystectomy bile leak were more common indications in the EIP compared to the non-EIP group (61% *versus* 53% and 7% *versus* 2% respectively). Choledocholithiasis (23% *versus* 28%), cholangitis (4% *versus* 0%) and gallstone pancreatitis (7% *versus* 11%) were more common indications in the non-EIP group. However, the observed differences in the ERCP indications did not reach statistical significance ( Table 2). Obstructive jaundice proved to be secondary to cancer in 2/8 patients in the EIP group. The rest were secondary to benign strictures.

*ERCP characteristics*

Failure to cannulate the common bile duct showed significant association with EIP in contrast to the non-EIP (46% *versus* 6%; P = 0.003). Interestingly, patients who underwent CBD dilatation experienced substantially lower rates of EIP (31% *versus* 67%: P = 0.0214). The presence of CBD dilatation and filling defects was less common among the EIP group (31% *versus* 46% and 15% *versus* 26% respectively) but with no statistical significance. We found that the most significant risk factor and predictor of duodenal perforation during ERCP was failure of cannulation with an odds ratio of 11.4 (P = 0.003).

*Diagnosis and management*

The majority of patients (11 patients, 84%) were diagnosed with EIP using CT scan while the remaining two patients (16%) were diagnosed during ERCP. One of the two patients developed extensive surgical emphysema during the operation while the other was diagnosed by directly visualizing the contrast extravasation through the duodenum. Of the patients diagnosed after the procedure, the most common symptom was abdominal pain (11 patients, 84%) with the remaining two patients (14%) presenting in the form of peritonitis.

Conservative treatment was preferred for eight patients (61%), while five patients (39%) were treated operatively; ( Table 3). Operatively treated patients were of a younger age and had less comorbidities such as hypertension and diabetes, which might have influenced the choice of management. Moreover, half of the conservatively-treated group had a history of abdominal surgery, namely laparoscopic cholecystectomy, compared to only one patient (20%) in the surgical group, which could also have affected the surgeon’s choice of treatment. Conservatively managed patients had more dilated CBD as opposed to the surgically treated (37% *versus* 20%). Papillotomy, dilatation of CBD and stone extraction were much more commonly performed in the conservatively treated group (100% *versus* 60%, 50% *versus* 0% and 37.5% *versus* 0%, respectively). Otherwise, there was no significant differences in ERCP characteristics between the surgical and the conservatively treated groups ( Table 3).

**Table 3.** Conservative vs. operative treatment: Patient and ERCP characteristics.

	Surgical group (n = 5)	Conservative group (n = 8)	P value
Sex - female (%)	3 (60%)	6 (75%)	^e^NS
Age (mean ± ^g^SD)	42.00 ± 14.24	53.00 ± 15.19	NS
Comorbidities			
^a^HTN - n (%)	2 (40%)	6 (75%)	NS
^b^IHD - n (%)	0 (0%)	0 (0%)	^f^NA
^c^DM - n (%)	0 (0%)	2 (25%)	NS
Previous abdominal surgery - n (%)	1 (20%)	4 (50%)	NS
Cholecystectomy - n (%)	1 (20%)	4 (50%)	NS
Relevant neoplasm - n (%)			NS
Pancreatic cancer - n (%)	0 (0%)	1 (12.5%)	NS
Cholangiocarcinoma - n (%)	1 (20%)	0 (0%)	NS
ERCP characteristics			
Dilated ^d^CBD - n (%)	1 (20%)	3 (37.5%)	NS
Filling defect - n (%)	1 (20%)	1 (12.5%)	NS
Papillotomy - n (%)	3 (60%)	8 (100%)	NS
CBD balloon dilatation - n (%)	0 (0%)	4 (50%)	NS
Stone extraction - n (%)	0 (0%)	3 (37.5%)	NS
Sludge extraction - n (%)	1 (20%)	3 (37.5%)	NS
Plastic stent placement - n (%)	2 (40%)	0 (0%)	NS
Pus - n (%)	0 (0%)	0 (0%)	NA
Failure of cannulation - n (%)	2 (40%)	4 (50%)	NS
Premature termination of procedure - n (%)	2 (40%)	4 (50%)	NS

^a^ HTN, hypertension.

^b^ IHD, ischemic heart disease.

^c^ DM, diabetes mellitus.

^d^ CBD, Common bile duct.

^e^ NS, not significant.

^f^ NA, not applicable.

^g^ SD, standard deviation.

*Treatment according to perforation type*

Overall, 75% of patients who had type I perforation were treated surgically whereas none of patients with type III or type IV perforation were operated. Type II perforation was divided between conservative treatment (two patients) and surgical management (one patient).

*Perforation type by treatment*

The majority (80%) of the surgically treated patients had type I perforation. Of those treated conservatively, three had type IV perforation, two had type II perforation, two had type I perforation and 1 had type III perforation ( Table 4).

**Table 4.** Conservative versus operative treatment: Perforation type, morbidity and mortality.

	Surgical group (n = 5)	Conservative group (n = 8)	P value
Perforation type			
Type I (free wall) - n (%)	4 (80%)	2 (25%)	NS
Type II (peri-vaterian) - n (%)	1 (20%)	2 (25%)	NS
Type III (distal ^a^CBD) - n (%)	0 (0%)	1 (12.5%)	NS
Type IV ( ^b^RP free air) - n (%)	0 (0%)	3 (37.5%)	NS
^c^LOS (days, mean ± SD)	24 ± 17.71	17.25 ± 11.69	NS
Morbidity			
^f^ICU admission- n (%)	3 (60%)	2 (25%)	NS
Duration of ICU admission (days, mean ± ^d^SD)	7.94 ± 4.58	3.00 ± 0.00	NS
Intra-abdominal abscess requiring drainage - n (%)	1 (20%)	1 (12.5%)	NS
^e^TPN/enteral feeding - n (%)	3 (60%)	4 (50%)	
Acute kidney injury - n (%)	0 (0%)	1 (12.5%)	NS
Pneumonia - n (%)	1 (20%)	0 (0%)	NS
Mortality - n (%)	1 (20%)	1 (12.5%)	NS

^a^ CBD, Common bile duct.

^b^ RP, retroperitoneal.

^c^ LOS, length of hospital stay.

^d^ SD, Standard deviation.

^e^ TPN, Total parenteral nutrition.

^f^ ICU, Intensive Care Unit.

### LOS, morbidity and mortality

Surgically treated patients stayed longer in the hospital with an average of 24 days compared to 17.25 days for the conservatively treated group ( Table 4). Additionally, ICU admission rates and the average LOS in the ICU were both higher among the surgically treated patients (60% *versus* 25% and 7.94 versus three days). Major complications were noted in slightly higher rates among the surgical group. Of those treated surgically, one patient (20%) suffered from an intra-abdominal abscess requiring drainage and another patient (20%) developed pneumonia. On the other hand, one patient (12.5%) among the conservatively treated patients had an intra-abdominal abscess requiring drainage and another patient (12.5%) sustained acute kidney injury. About half of the patients in both groups received enteral or parenteral feeding at some point of their hospital stay. The overall mortality rate was 15% with a slightly higher rate in the surgical group (20% *versus* 12.5%).

## Discussion

In this study, we investigated the incidence rate of duodenal perforation among patients undergoing ERCP and compared patients’ and procedure’s characteristics to a control group. Our results revealed that perforations occurred in a comparable rate (1.34%) to published series. Our study also disclosed that failure to cannulate the CBD was the main predictor of perforation during ERCP.

The reported incidence of ERCP related perforations varied between 0.11% and 2.4%. ^6^^,^^7^ In one large analysis of 21 prospective surveys of post-ERCP complications in adults (16,855 patients) between 1977-2006, 101 patients experienced perforation (0.60%). The mortality rate in that study was 9.9%. ^3^ In another review of 18 (mainly retrospective) studies conducted between 2000-2014, the incidence rate of EIP was 0.39%, with an overall mortality of 7.8%. ^8^ Surprisingly, published numbers did not seem to decline significantly over the last 20 years as ERCP became more popular and widely available.

Published data revealed that the incidence rate of ERCP perforations corresponds with volume of cases at each institution, as highes-volume centers (which perform more than 1000 ERCPs per year) reported lower incidence rates (Bill *et al.*, 0.44%, Jin *et al.*, 0.27%, Kim J *et al.*, 0.61%, Howard *et al.*, 0.6%, Dubecz *et al.*, 0.09%), ^9^^–^^13^ compared to the lower volume ones (less than 500 ERCPs per year) (Turner *et al.*, 2.4%, Stapler *et al.*, 1%, Miller *et al.*, 1.65%, Rabie *et al.*, 1.67%, Koc *et al.*, 0.94%). ^5^^,^^6^^,^^14^^–^^16^ The correlation between incidence of perforation and volume of ERCP cases needs further analysis to elaborate statistical significance. These observations, however, indicate that ERCP remains an invasive procedure which may carry significant morbidity and mortality, even in skilled hands or at high volume units.

“Difficult cannulation” is a term employed to describe failure to gain access into the bile duct by the conventional cannulation technique. Factors that may contribute to this difficulty include presence of periampullary diverticula, altered anatomy and bulky papilla. In many studies, this situation has been defined as a leading cause of perforation. ^17^ The risk is highest as precut papillotomy (or sphincterotomy) is attempted by the endoscopist to overcome this challenge. In Vezakis *et al.*’s review, endoscopic sphincterotomy was responsible for 41% of perforations. ^8^ Another study by Stapfer and colleagues found that cannulation of the ampulla was considered difficult by the endoscopist in 10 of 14 patients who had perforations (out of 1413 ERCPs). ^5^ In their review of 21 prospective reports of ERCP complications, Andriulli *et al.*, found that the overall complication rate was significantly higher whenever therapeutic interventions were utilized during ERCP. ^3^ Complications were also significantly higher in studies with a precut sphincterotomy rate of over 10%. Fifty years after the advent of therapeutic ERCP, difficult cannulation is still a frequently encountered condition, which may – although rarely – predispose to perforation and subsequent morbidity and death.

There appears to be a consensus on electing surgery for the treatment of free duodenal wall perforations (type I), which commonly present with signs of peritonitis. ^12^^,^^18^^–^^20^ In our group of patients with perforation, surgery was required in 5/13(38%), 4/5 of the operated patients had type I (free wall) perforation. Surgical intervention is conducted in higher risk injuries that are more likely to progress into sepsis, which explains – in part – the higher morbidity observed in this subgroup. Because duodenal or duct wall defect identification during surgery might be very challenging, especially if diagnosis is delayed (>6 hours post ERCP), ^21^^,^^22^ surgery can be limited to perform proper drainage and debridement of unhealthy tissue, which can reduce the risk of systemic manifestations and sepsis. Diversion surgeries (Roux-en-Y bypass) have been reported in management of large duodenal perforations. ^5^ However, in ERCP most perforations are small, unless caused by the scope itself.

Endoscopic repair of duodenal perforations, particularly small defects(<10mm) that are recognized during ERCP has been reported. ^23^^,^^24^ The latter development of endoscopic clipping (through-the-scope clips), suturing and closure devices (ligation band, fibrin glue, and endo-loops) as well as covering luminal stents has made endoscopy more efficient in treating injuries similar to perforations and bleeding. ^25^^–^^31^ With the growing body of evidence supporting the use of these techniques for the management of ERCP complications, endoscopist expertise, perforation type and diameter remain important predictors of the outcomes.

In other types of perforation, *i.e.* types II, III and IV, the injury is less likely to manifest as peritonitis, but rather as retroperitoneal (and to a less extent intraperitoneal) fluid or air accumulation. Conservative measures in such circumstances vary from simply restricting oral intake with parenteral nutrition, hydration and coverage with broad spectrum antibiotics to percutaneous drainage of collections under ultrasound or CT guidance.

It is critical to recognize if leakage has stopped after the endoscopy or is still ongoing, as the patient may have experienced transient fluid extravasation during the procedure due to duct or duodenal wall puncture. The presence of enlarging pockets of pus or fluid collections, especially in the retroperitoneal space, does not necessarily indicate an active leakage, which can usually be excluded utilizing CT scan with water soluble contrast (Gastrografin), or fluoroscopic imaging (meal). This may instead indicate inadequate drainage or persistent infection that mandates repositioning of the drain, upgrading the size of the drain or placing another drain. It may also be helpful to consider adding antifungals after submitting samples for cultures.

The following are literature-based guidelines which can be driven to conclude when and how to intervene in patient with suspected or proved ERCP-induced perforation:

• Suspicion is raised whenever cannulation is difficult, the threshold for obtaining a post ERCP CT scan to exclude perforation has to be lowered, since early detection may improve outcomes.
• Active intraperitoneal contrast extravasation from duodenal wall on CT scan is considered a reasonable indication for prompt operative exploration.
• The main target of surgical exploration is to control sepsis by drainage of accumulated fluids. Repair of the defect, if identified, is another target with special attention to prevent occlusion of the ducts.
• The presence of free fluids inside the peritoneum or in the retroperitoneal space is an indication for drainage; this can be achieved by interventional radiology (IR) under CT scan or ultrasound guidance, or by surgery if IR service is not available or the collection is not accessible.
• Most bile duct perforations can be managed by internal biliary stents, along with drainage of any collection. External nasobiliary drainage is considered a valid alternative for internal stenting.
• Failure of non-operative measures, which can be defined as the persistence of abdominal sepsis (significant pain, tenderness and ileus), as well as fever and continuous elevation of inflammatory markers (leukocytes, CRP, ESR, among others), indicates prompt conversion into surgical approach.
• Prolonged restriction of oral intake may not be of any help if there is no evidence of an ongoing leakage.
• ERCP perforation remains an event that has to be approached by both surgeons and endoscopists.

This study had some limitations. Firstly, it was a retrospective study. Secondly, the sample size of patients with EIP was relatively small to evaluate risk factors with statistical significance. Thirdly, we assume that a small group of patients with type IV perforation may have not been detected, due to lack of abdominal or systemic manifestations.

In conclusion, fifty years after introduction of ERCP for therapy, it remains an invasive procedure with certain complications that carry significant morbidity and mortality, even in skilled hands or at high volume units. Selection of patients for ERCP must be strict, it has to be done for therapeutic indications. Difficult cannulation is still a condition that is frequently encountered, and considered the main risk factor for perforation. Early diagnosis and appropriate surgical or percutaneous drainage yield favorable outcomes.

## Author contributions

AM: investigation, methodology and project administration, ZM: resources, software, TM: software, analysis, HM: software, supervision, AH: methodology, resources, KJ: investigation, MH: methodology, NF: investigation, SS: resources, DA: resources, supervision, JF: methodology. All authors had substantial contribution to conception and design, interpretation of data, drafting, and revising the article. They all approved the final manuscript and agreed to be accountable for all aspects of the work.

## Data Availability

Data supporting the study’s findings are available upon request from the corresponding author, Dr. Abdel Rahman A. Al Manasra, (request by email: Aaalmanasra@just.edu.jo). The data are not made public because they contain information that could jeopardize the privacy of research participants.
