# Supplementary material for: ERCP-induced perforation: review and revisit after half a century
Source: F1000Res. 2024 Mar 15;12:612. Originally published 2023 Jun 6. [Version 2] doi: 10.12688/f1000research.129637.2 (PMC11255546; doi:10.12688/f1000research.129637.2)
Supplement: Peer review history

# Reviewer report: Reviewer response for version 2

**DOI:** 10.5256/f1000research.163615.r284158

**Published:** 2024-07-17

**Authors:** Bugra Tolga Konduk

**Affiliations:** 1 Gaziantep University, Gaziantep, Turkey

I have read the review which I think had been given a lot of effort during preparation. However, I have some criticisms and comments.

 "Did the patients with EIP have adjacent diverticula, intradiverticular papilla , or were the patients’ papilla morphology assessed and classified? This is important because this anomaly may cause difficult cannulation and inadvertent injury to papilla resulting in perforation? This data may be added if available.

 1) Haraldsson, E. et al. (2019 [Ref - 1]). DOI: https://doi.org/10.1016/j.gie.2019.07.014

 2)Tari, E. et al. (2024 [Ref - 2]). DOI: https://doi.org/10.1038/s41598-024-57758-9"

 EIP is a complication due to mechanical maneuvers and interventional techniques during ERCP, so being hypertensive or diabetic should not be considered as a risk factor for perforation but rather be regarded as an important risk factor in the post-procedure period of patient care, should there be a perforation during ERCP. I believe the manuscript should be corrected in this aspect, if the authors agree.

 The precut papillotomy rate was reported as 85% for the EIP and this rate is quite high, why do the operators prefer this much high rate of precut papillotomy? Do the operators prefer other options such as double guide cannulation, trans-pancreatic sphincterotomy,  etc.,  before commencing to a more hazardous technique such as a free-hand precut papillotomy? Is the rate of precut papillotomy significantly lower in the  non-EIP patients when compared to EIP patients? If so, precut papillotomy utilization should be regarded as the most important risk factor for EIP, concerning your manuscript. This may be added to the manuscript, if this makes sense for the authors too.

 I suggest that the methodology for statistical analysis should be reviewed by either an independent or the attendant statistician of the journal.

 I have read this submission. I believe that I have an appropriate level of expertise to confirm that it is of an acceptable scientific standard, however, I have a number of small changes to the article, or specific, sometimes more significant revisions.

Is the work clearly and accurately presented and does it cite the current literature?

Yes

If applicable, is the statistical analysis and its interpretation appropriate?

I cannot comment. A qualified statistician is required.

Are all the source data underlying the results available to ensure full reproducibility?

Yes

Is the study design appropriate and is the work technically sound?

Yes

Are the conclusions drawn adequately supported by the results?

Yes

Are sufficient details of methods and analysis provided to allow replication by others?

Yes

Reviewer Expertise:

Gastroenterohepatology, pancreatobiliary diseases, interventional endoscopy, ERCP

I confirm that I have read this submission and believe that I have an appropriate level of expertise to confirm that it is of an acceptable scientific standard, however I have significant reservations, as outlined above.

## References

[1] : Macroscopic appearance of the major duodenal papilla influences bile duct cannulation: a prospective multicenter study by the Scandinavian Association for Digestive Endoscopy Study Group for ERCP. *Gastrointest Endosc* .2019;90(6) : 10.1016/j.gie.2019.07.014 957-963 10.1016/j.gie.2019.07.014 31326385

[2] : Morphology of the papilla can predict procedural safety and efficacy of ERCP-a systematic review and meta-analysis. *Sci Rep* .2024;14(1) : 10.1038/s41598-024-57758-9 7341 10.1038/s41598-024-57758-9 38538734 PMC10973369

# Reviewer report: Reviewer response for version 2

**DOI:** 10.5256/f1000research.163615.r256434

**Published:** 2024-03-21

**Authors:** Budhi Ida Bagus

**Affiliations:** 1 Sebelas Maret University, Surakarta, Indonesia

No more revision needed.

Is the work clearly and accurately presented and does it cite the current literature?

Yes

If applicable, is the statistical analysis and its interpretation appropriate?

Yes

Are all the source data underlying the results available to ensure full reproducibility?

Yes

Is the study design appropriate and is the work technically sound?

Yes

Are the conclusions drawn adequately supported by the results?

Yes

Are sufficient details of methods and analysis provided to allow replication by others?

Yes

Reviewer Expertise:

Gastrointestinal Cancer, Colorectal Cancer, Acute Care Surgery, Immune-Oncology , Chemotherapy, Enhanced Recovery After Surgery

I confirm that I have read this submission and believe that I have an appropriate level of expertise to confirm that it is of an acceptable scientific standard.

# Reviewer report: Reviewer response for version 1

**DOI:** 10.5256/f1000research.142334.r235317

**Published:** 2024-02-15

**Authors:** Budhi Ida Bagus

**Affiliations:** 1 Sebelas Maret University, Surakarta, Indonesia

Good written review manuscript.  It might be better if you can described the criteria of endoscopist who performed the ERCP procedures (if possible).

 Please add the information whether the ERCP-induced perforation cases were occurred in the first or the second procedure of ERCP?

 Were there any information about the duration of each ERCP procedures which associated with perforation?

Is the work clearly and accurately presented and does it cite the current literature?

Yes

If applicable, is the statistical analysis and its interpretation appropriate?

Yes

Are all the source data underlying the results available to ensure full reproducibility?

Yes

Is the study design appropriate and is the work technically sound?

Yes

Are the conclusions drawn adequately supported by the results?

Yes

Are sufficient details of methods and analysis provided to allow replication by others?

Yes

Reviewer Expertise:

Gastrointestinal Cancer, Colorectal Cancer, Acute Care Surgery, Immune-Oncology , Chemotherapy, Enhanced Recovery After Surgery

I confirm that I have read this submission and believe that I have an appropriate level of expertise to confirm that it is of an acceptable scientific standard, however I have significant reservations, as outlined above.

# Response

**Published:** 2024-03-10

**Authors:** Zaid Mesmar

**Affiliations:** Jordan University of Science and Technology, Jordan

Dear sir,

 Thank you very much for the time spent reviewing our manuscript. We have answered all your valuable comments one by one .

 Again, we highly appreciate your effort and we hope our response will meet your expectations.

 1- The criteria of endoscopist who performed the ERCP procedures are added to the text ( please see the result section , first paragraph)  : board certified gastroenterologist, licensed to perform endoscopy after training in an accredited structured ERCP program.

 2- The ERCP-induced perforation cases were occurred in the first time , this has also been clarified in the result section first paragraph. 

 3- Unfortunately , the duration of the procedure was not documented in the ERCP reports , neither stored on the patients electronic files.

# Reviewer report: Reviewer response for version 1

**DOI:** 10.5256/f1000research.142334.r238886

**Published:** 2024-02-05

**Authors:** Vaibhav Wadhwa, Prateek Harne

**Affiliations:** 1 University of Texas Health Science Center, Texas, USA

Thank you for submitting your detailed work to this journal. Authors present data on ERCP-induced perforation which serves as a helpful guide for gastroenterologists. There are some major revisions that are suggested:

 Patient demographic table.

 For standardizing the impact of comorbidities on the outcomes, it is recommended to use a standardized and validated comorbidity index such Charleson Comorbidity Index (CCI) (Ref 1, Ref 2 ). This will help in standardizing the comorbidity burden.

 Additionally, a sub-group analysis to see correlation with higher CCI to outcome (specifically between conservative and operative arm) can provide more information on case selection for intervention and increase generalizability of this study.

 Failure of cannulation:

 Since two patients (of the 13) were found to have a perforation during the procedure, it is assumed that the procedure was prematurely terminated. It should be considered to remove them from the analysis of ‘failed cannulation’ as that instills bias. In other words, in these two patients, perforation led to failed cannulation and not the other way around.

 Length of procedure and length to cannulation:

 It would be helpful to know if there was a difference in the two arms when it came to (1) total length of the procedure and (2) time to cannulation. These parameters are linked to post-ERCP pancreatitis and it would be useful to include them in analysis to see if they correlate with perforations (Ref 3).

Is the work clearly and accurately presented and does it cite the current literature?

Yes

If applicable, is the statistical analysis and its interpretation appropriate?

Partly

Are all the source data underlying the results available to ensure full reproducibility?

Partly

Is the study design appropriate and is the work technically sound?

Partly

Are the conclusions drawn adequately supported by the results?

Partly

Are sufficient details of methods and analysis provided to allow replication by others?

Partly

Reviewer Expertise:

Interventional Endoscopy

We confirm that we have read this submission and believe that we have an appropriate level of expertise to state that we do not consider it to be of an acceptable scientific standard, for reasons outlined above.

## References

[1] : A new method of classifying prognostic comorbidity in longitudinal studies: development and validation. *J Chronic Dis* .1987;40(5) : 10.1016/0021-9681(87)90171-8 373-83 10.1016/0021-9681(87)90171-8 3558716

[2] : Charlson Comorbidity Index: A Critical Review of Clinimetric Properties. *Psychother Psychosom* .2022;91(1) : 10.1159/000521288 8-35 10.1159/000521288 34991091

[3] : Difficult Biliary Cannulation from the Perspective of Post-Endoscopic Retrograde Cholangiopancreatography Pancreatitis: Identifying the Optimal Timing for the Rescue Cannulation Technique. *Gut Liver* .2021;15(3) : 10.5009/gnl19304 459-465 10.5009/gnl19304 32000469 PMC8129666

# Reviewer report: Reviewer response for version 1

**DOI:** 10.5256/f1000research.142334.r177536

**Published:** 2023-08-29

**Authors:** Hazem Hammad

**Affiliations:** 1 Division of Gastroenterology and Hepatology, Department of Medicine, University of Colorado, Aurora, Colorado, USA

Thanks for submitting this excellent research project. 

 Page #4 and table 2: Need to specify precut papillotomy vs papillotomy. Also need to look into how many patients actually received sphincterotomy (with length) and balloon sphincteroplasty (with diameter).

 Page #4: I am not why the status of having HTN or DM was looked at. Was this relevant?

 Page #5, Table 2.:

- For the obstructive jaundice, were these all due to underlying malignancy such as HOP cancer? worth elaborating more. 
- For "CBD balloon dilation", did this mean CBD stricture balloon dilation or balloon sphincteroplasty? 

Page 6 and table 3: Please comment on stent placement (plastic/metal) in cases of EIP. 

 Page #6: Worth mentioning if any patient had post ERCP pancreatitis and whether that could have affected the outcome. 

 Table #4: I am not sure that comparing surgical and conservative groups help given that the main difference is likely the severity/type of perforation so may be comparing the outcomes of different types of perforation would have been more helpful. 

 Page 8: "In other types of perforations i.e types I, II and IV"...was it meant to be "type II, III and IV?

Is the work clearly and accurately presented and does it cite the current literature?

Yes

If applicable, is the statistical analysis and its interpretation appropriate?

Yes

Are all the source data underlying the results available to ensure full reproducibility?

Yes

Is the study design appropriate and is the work technically sound?

Partly

Are the conclusions drawn adequately supported by the results?

Partly

Are sufficient details of methods and analysis provided to allow replication by others?

Partly

Reviewer Expertise:

Advanced endoscopic procedures

I confirm that I have read this submission and believe that I have an appropriate level of expertise to confirm that it is of an acceptable scientific standard, however I have significant reservations, as outlined above.

# Response

**Published:** 2023-11-09

**Authors:** Zaid Mesmar

**Affiliations:** Jordan University of Science and Technology, Jordan

Dear sir,

 Thank you very much for the time spent reviewing our manuscript.

 We have answered all your valuable comments one by one in the following table.

 Again, we highly appreciate your effort and we hope our response will meet your expectations.

 1-The term papillotomy used in Table 2 refers to precut papillotomy, this has been discussed in the text following Table 2, “ERCP characteristics” paragraph.

 2-Unfortunately, the length of the sphincterotomy and the diameter of the balloon were not included in the ERCP reports for most patients. So, this part was skipped in spite of the value that could be added to the analysis of outcomes.

 3-The status of having HTN and DM, especially when uncontrolled, contributes significantly to the  morbidity and mortality in hospitalized patients or in patients who undergo invasive procedures.

 It may also impact the optimal management plan for patients who sustain injury.

 4-Obstructive jaundice proved to be secondary to cancer in 2/13 patients in the EIP group. The rest were secondary to benign strictures.

 5-“CBD balloon dilatation” refers to CBD stricture balloon dilatation. This will be clarified in Table 2

 6-The stent type used was plastic in EIP, the comment will be added to Table 3.

 7- Correct, post-ERCP pancreatitis may affect outcomes in patients with EIP. However, it was not tracked in this analysis which focused on the types of perforation and outcomes in light of management choice, we may conduct a sub-analysis after revisiting patients’ data and gathering necessary details in future work.

 8- The option of treatment correlates with the type of perforation, however, the outcomes have been discussed from a different perspective, which is the mode of treatment. The question raised initially was: do more invasive treatment options carry worse outcomes? The only approach to answer this was to review outcomes in the light treatment method.

 9- Yes, sir, it was meant to be type II. III and IV,

 The editor is contacted so that the error will be corrected in the original manuscript.
